# Prevalence of Mental Illness and Mental Health Care Use Among Police Officers

**DOI:** 10.1001/jamanetworkopen.2020.19658

**Published:** 2020-10-07

**Authors:** Katelyn K. Jetelina, Rebecca J. Molsberry, Jennifer Reingle Gonzalez, Alaina M. Beauchamp, Trina Hall

**Affiliations:** 1 Department of Epidemiology, Human Genetics, and Environmental Sciences, The University of Texas Health Science Center at Houston School of Public Health, Dallas; 2 Meadows Mental Health Policy Institute, Dallas, Texas; 3 Psychological Services, Dallas Police Department, Dallas, Texas

## Abstract

**Importance:**

Limited literature has characterized patterns of mental illnesses and barriers in seeking mental health care among police officers.

**Objectives:**

To assess the prevalence of mental illness (diagnosis) and symptoms of mental illness, evaluate the characteristics of officers interested in seeking mental health care, and characterize perceptions of mental health care use.

**Design, Setting, and Participants:**

This survey study was conducted among officers at a large police department in Dallas–Fort Worth, Texas. Focus group sessions were conducted from April 1, 2019, to November 30, 2019, and the survey was conducted from January 1 to February 27, 2020. A total of 446 sworn, employed patrol officers who were present during the recruitment briefing were eligible to participate in surveys and focus groups.

**Main Outcomes and Measures:**

Officers reported lifetime or current diagnosis of depression, anxiety, and posttraumatic stress disorder, as well as current mental health symptoms (using validated screeners of depression, anxiety, posttraumatic stress disorder, and suicidal ideation or self-harm) and mental health care use in the past 12 months. Focus group data were collected to contextualize mental health care use. Logistic regression analyses were used for quantitative data, and focus groups were iteratively coded by 4 coders using inductive and deductive thematic identification.

**Results:**

Of the 446 officers invited to participate, 434 (97%) completed the survey (mean [SD] age, 37 [10] years; 354 [82%] male; 217 White [50%]). Of these officers, 19 (17%) had sought mental health care services in the past 12 months. A total of 54 officers (12%) reported a lifetime mental health diagnosis, and 114 (26%) had positive screening results for current mental illness symptoms. Among officers with positive screening results, the odds of interest in using mental health services was significantly higher for officers with suicidal ideation or self-harm than for those who did not (adjusted odds ratio, 7.66; 95% CI, 1.70-34.48). Five focus groups were conducted with 18 officers and found 4 primary barriers in accessing mental health services: (1) inability to identify when they are experiencing a mental illness, (2) concerns about confidentiality, (3) belief that psychologists cannot relate to their occupation, and (4) stigma that officers who seek mental health services are not fit for duty.

**Conclusions and Relevance:**

The study found that although few officers were seeking treatment, they were interested in seeking help, particularly those with suicidal ideation or self-harm. Additional interventions appear to be needed to systematically identify and refer officers to health care services while mitigating their concerns, such as fear of confidentiality breach.

## Key Points

**Question:**

What is the prevalence of mental illness and mental health care use among police officers at a large, urban police department?

**Findings:**

In this survey study of 434 police officers, 12% had a lifetime mental health diagnosis and 26% reported current symptoms of mental illness. Of these officers, 17% had sought mental health care services in the past 12 months, but officers reported interest in help if a few key concerns were met, including confidentiality assurance.

**Meaning:**

The findings suggest that routine mental health screening may be needed in law enforcement agencies to systematically identify and refer officers to mental health care services.

## Teaser

This survey study assesses the prevalence of mental illness and mental health care use among police officers at a large, urban police department in the US.

## Introduction

Evidence suggests that exposure to law enforcement work is associated with increases in many forms of stress, including physical, psychosocial, and anticipatory stress.^1,2,3^ Officers are exposed to traumatic calls for service on a daily basis, including child abuse, domestic violence, car crashes, and homicides. Repeated exposure to these stressors and events may be associated with development of mental illnesses, such as anxiety, depression, somatization, posttraumatic stress disorder (PTSD), and burnout.^4,5,6,7,8,9^ In 2019, twice as many officers died of suicide compared with dying in the line of duty.^10^ Within the Buffalo, New York Police Department, the odds of committing suicide were 8.4 times higher among active duty officers compared with retired officers.^11^

Although the prevalence of mental illness among officers has been documented,^12,13,14,15^ limited literature has characterized patterns and barriers of mental illnesses and described characteristics of officers who express interest in seeking help. Fox et al^16^ conducted a study among 150 officers in a suburban police department in the northeastern US. They found that 46% of officers with a mental illness ever sought mental health care. However, this study was conducted more than 10 years ago, and the department was in a suburban area, where occupational exposures and associated triggers for mental illness may be different compared with a city. Understanding the current barriers to mental health care use is critical in developing future interventions that can increase mental health care uptake by targeting officer needs. The purpose of this survey study was to assess the prevalence of lifetime mental illness diagnoses and current symptoms of mental illness among police officers, evaluate characteristics of police officers who expressed interest in seeking mental health care, and characterize officer perceptions of mental health care use.

## Methods

### Study Design

We used a convergent, iterative, survey design to accomplish the main aims of this study. This study was approved by the Center for the Protection of Human Subjects at The University of Texas Health Science Center at Houston. All participants provided written informed consent. Officers were given the choice to provide their badge number or not; 60% of them opted to provide their identify for follow-up analyses. This study followed the American Association for Public Opinion Research (AAPOR) reporting guideline.

### Study Population

In 2019, the Dallas Police Department (DPD) was the ninth largest police department in the US and employed approximately 3300 officers, with 1400 patrol officers answering 911 calls to more than 1.32 million civilians. There are 7 patrol divisions within the DPD, and each patrol division is assigned geographic boundaries. Eligible participants for this study were sworn, currently employed DPD patrol officers and had the opportunity to participate in both the surveys and the focus groups.

### Data Collection

#### Surveys

Patrol officers met for a routine briefing during the first 30 minutes of their shift. The purpose of these briefings was to take attendance, brief the officers on crime clusters and criminals in the area, and describe unfinished business from previous shifts and administrative matters. For this study, 1 of us (K.K.J.) attended briefings across all divisions and 3 shifts, which totaled 46 briefings, from January 1 to February 27, 2020. At each briefing, 1 of us (K.K.J.) explained the purpose of the study and invited officers to use a quick response (QR) code to complete a 15-minute survey on their smartphone. Of the 446 officers invited to participate, 434 (97%) consented to participate. The total sample of 434 participants represented 31% of the total patrol population at the DPD (n = 1413). Personnel records were abstracted to describe the demographic characteristics of the entire patrol officer population.

Focus groups were conducted from April 1 to November 30, 2019. A total of 18 patrol officers were recruited to participate in 5 focus groups. Similar to survey recruitment, 1 of us (K.K.J.) attended select details to recruit officers. During these briefings, 1 of us (K.K.J.) described the project and explained that participation was voluntary and a decision to not participate would have no ramifications on the officer’s employment status. Officers who were interested in participating were instructed to provide contact information on a paper log at the briefing or by email (to K.K.J.). One of us (K.K.J.) scheduled all focus groups 1 week after recruitment, and recruitment ended when results reached saturation. Officers were provided an incentive of $30 per hour in the form of a gift card in exchange for their time.

Focus groups were conducted on site at patrol substations in a private conference room 2 hours before officers’ shifts. Before the focus group began, officers completed a written informed consent form and a brief, 8-item demographic survey. The semistructured focus groups lasted approximately 1.5 hours each and began with a general discussion about the project. During the focus groups, officers were asked to discuss how repeated stress and coping mechanisms varied, depending on length of time on the job, decompression techniques used during and after a shift, and mental health care use.

### Survey Measures

#### Mental Illness Diagnosis

Officers self-reported lifetime diagnoses of depression, anxiety, and PTSD. If officers reported having been diagnosed with a condition, they were asked when they were diagnosed and whether they were currently receiving treatment for the condition.

#### Mental Illness Symptom Screening

Officers not currently diagnosed with a particular mental illness completed the following validated measures: Patient Health Questionnaire 2 (PHQ-2), Generalized Anxiety Disorder 2 (GAD-2), and Primary Care Posttraumatic Stress Disorder for *Diagnostic and Statistical Manual of Mental Disorders* (Fifth Edition) (PC-PTSD-5). Officers were considered to have positive screening results if they had a cut-off value of 3 or more (PHQ-2: sensitivity, 83%; specificity, 92%^17^; PC-PTSD-5: sensitivity, 93%; specificity, 85%^18^; and GAD-2: sensitivity, 86%; specificity, 83%^19^). Officers were also asked about suicide ideation or self-harm as a part of the PHQ screener: “Over the last 2 weeks, how often have you been bothered by thoughts that you would be better off dead or of hurting yourself in some way?”

Mental health care use was measured using 2 items: (1) “Over the past 12 months, which type of healthcare provider have you visited?” with 1 of the response options being mental health services. If the respondent selected mental health services or responded affirmatively to the question, (2) “During the past 12 months, have you seen or talked to a mental health professional such as a psychologist, psychiatrist, psychiatric nurse, counselor, clinical social worker about your health?” officers were classified as having used mental health care in the past year.

#### Interest in Mental Health Care Services

At the end of the survey, officers with positive screening results for depression, anxiety, PTSD, or suicidal ideation or self-harm symptoms were asked, “Given your responses, you may benefit from mental health services. Are you interested in getting help?”

#### Confounders

Officers were also asked to provide demographic characteristics, including age (years), gender (male, female, or other), race/ethnicity (non-Hispanic White, non-Hispanic Black, Hispanic, or other or more than 1 race/ethnicity), marital status (married; divorced, widowed, or separated; never married; or unmarried couple), and educational level (high school diploma, some college or technical school, college graduate, or master’s graduate or higher). We also collected military experience (years in military and military device C [combat merit], R [remote merit], or V [valor]) because of the association between military status and law enforcement outcomes.^20^ In addition, we collected employment history, including length of service (years), current shift (12 pm to 7 am, 7 am to 3 pm, 3-11 pm, or other), and rank (patrol officer, sergeant, or lieutenant or higher). All confounders were included in multivariable models given their association with officer wellness.^9,20,21,22,23,24^

### Statistical Analysis

#### Quantitative Analysis

Univariate statistics (tabulations, frequencies, means, and SDs) were used to describe the study population, the prevalence of mental illness diagnoses, and the rate of positive screening results for mental illness symptoms. We used bivariate logistic regression to identify officer-level characteristics associated with a lifetime mental illness diagnosis or a positive screening result for mental illness symptoms. Because significant associations were detected, logistic regression models were expanded to include sociodemographic and occupational variables that have been associated with officer wellness. Furthermore, bivariate and multivariable logistic regression models were used to evaluate the association between a positive screening result and interest in mental health care services. All hypothesis tests were 2-sided, and we used an a priori α = .05. Quantitative analyses were conducted using SAS statistical software, version 9.4 (SAS Institute Inc).^25^

#### Qualitative Analysis

All focus groups were audio recorded and professionally transcribed. A multidisciplinary team used a 4-step approach to analyze qualitative data.^26^ First, the research team (K.K.J., R.J.M., A.M.B., and others) read transcripts collected from each focus group to develop a deeper understanding of the group discussion. Through this process, a deductive codebook was created to label text. We used these codes in group analysis sessions until we reached stability. Second, text was coded by the research team. We grouped emerging findings into categories of themes using an immersion-crystallization approach,^27^ which included inductive thematic identification. Third, transcripts were read by a second coder, and coding inconsistencies were discussed and resolved by consensus. Finally, we considered how our findings related to the literature. NVivo software, version 12.0 (QSR International Pty Ltd) was used for all coding, organization, and data reduction.

## Results

### Surveys

Of the 446 officers invited to participate, 434 officers (97%) participated in the survey (mean [SD] age, 37 [10] years; 354 [82%] male; 217 White [50%]). Table 1 gives the sample description stratified by survey or focus group participation compared with the general DPD patrol officer population. A total of 237 (55%) were married, 222 (51%) graduated college, and 99 (24%) were military veterans. Older and more experienced officers were more likely to participate in the survey compared with the general officer population. Race/ethnicity also was significantly different among those who participated in surveys compared with their representation in the general population of officers. Overall, 54 officers (12%) reported a mental illness diagnosis in their lifetime, and 114 (26%) had positive screening results for mental illness symptoms in the past 2 weeks.

**Table 1.** Characteristics of the Study Sample^a^

Characteristic	Survey group (n = 434)	Focus groups (n = 18)	DPD patrol officers (n = 1413)
Age, mean (SD), y	37 (10)	38 (11)	35 (9)
Gender			
Male	354 (82)	17 (94)	1155 (82)
Female	68 (16)	1 (6)	258 (18)
Other	2 (0.5)	0	0
Missing	10 (2)	0	0
Race/ethnicity			
Non-Hispanic			
White	217 (50)	10 (56)	604 (43)
Black	62 (14)	4 (22)	374 (26)
Hispanic	112 (26)	4 (22)	379 (27)
Other or >1	22 (5)	0	56 (4)
Missing	21 (5)	0	0
Marital status			
Married	237 (55)	9 (50)	NR
Divorced, widowed, or separated	61 (14)	4 (22)	NR
Never married	86 (20)	3 (17)	NR
A member of an unmarried couple	31 (7)	2 (11)	NR
Missing	19 (4)	0	NR
Education			
High school diploma	17 (4)	1 (6)	NR
Some college or technical school	155 (36)	6 (33)	NR
College graduate	222 (51)	10 (56)	NR
Masters graduate or higher	21 (5)	1 (6)	NR
Missing	19 (4)	0	NR
Military experience	99 (24)	2 (11)	335 (24)
Military medal C, R, or V^b^	59 (14)	NR	NR
Time in military, y			
<3	7 (2)	NR	NR
3-7	46 (10)	NR	NR
8-11	28 (6)	NR	NR
12-15	5 (1)	NR	NR
>15	13 (3)	NR	NR
Length of service, mean (SD), y	11 (9)	13 (10)	8 (8)
Shift			
12-7 am	114 (26)	9 (50)	NR
7 am to 3 pm	140 (32)	7 (39)	NR
3-11 pm	136 (31)	2 (11)	NR
Other	24 (6)	0	NR
Missing	20 (5)	0	NR
Rank			
Patrol officer	366 (84)	NR	1413 (100)
Sergeant, lieutenant, or higher	44 (10)	NR	0
Missing	24 (6)	NR	0
Lifetime mental illness diagnoses^c^	54 (12)	NR	NR
Positive screen for mental illness symptoms in the past 2 wk^d^	114 (26)	NR	NR

Abbreviations: DPD, Dallas Police Department; NR, not reported.

^a^ Data are presented as number (percentage) of study participants unless otherwise indicated.

^b^ A C device indicates combat merit; R device, remote merit; and V device, valor.

^c^ Depression, anxiety, or posttraumatic stress disorder.

^d^ Positive screening result for depression, anxiety, posttraumatic stress disorder, and/or suicidal ideation/self-harm.

### Focus Groups

Among the officers who participated in focus groups, the mean (SD) age was 38 (11) years, 17 (94%) were male, 10 (56%) were non-Hispanic White, 9 (50%) were married, 10 (56%) were college graduates, and 2 (11%) reported military experience. More experienced officers were more likely to participate in focus groups compared with the general officer population.

Table 2 gives the prevalence of lifetime mental illness diagnoses and current positive screening results for mental illness symptoms in the past 2 weeks. Overall, 54 officers (12%) reported a lifetime mental illness diagnosis, and among those, 26 (48%) reported a current diagnosis. Overall, 114 officers (26%) screened positive for mental illness symptoms in the past 2 weeks. Among those with positive screening results, PTSD screening rates were the highest (69 [61%]) followed by depression symptoms (50 [44%]).

**Table 2.** Prevalence of Mental Illness Diagnoses and Positive Screening Results for Mental Illness Symptoms Among 434 Police Officers

Variable	Lifetime diagnosis (n = 54)^a^		Positive screening result in past 2 wk (n = 114)^b^
	Not current (n = 28)	Current (n = 26)	
Anxiety	10 (36)	26 (100)	39 (34)
Depression	11 (39)	11 (42)	50 (44)
PTSD	19 (68)	0	69 (61)
Suicide ideation or self-harm	0	0	21 (18)
Sought mental health services in past 12 mo	9 (32)	9 (35)	19 (17)

Abbreviation: PTSD, posttraumatic stress disorder.

^a^ Depression, anxiety, or PTSD.

^b^ Positive screening result for depression, anxiety, PTSD, and/or suicidal ideation or self-harm symptoms in past 2 weeks.

Table 2 also gives the prevalence of seeking mental health care in the past 12 months. Among officers with a current mental health diagnosis, 9 (35%) sought services in the past 12 months. Among those who screened positive for mental health symptoms, 19 (17%) reported using services in the past 12 months.

Table 3 gives the adjusted association between officer characteristics, lifetime diagnoses, and positive screening results. After adjustment for confounders, the odds of a lifetime mental illness diagnosis were significantly higher among female officers compared with male officers (adjusted odds ratio [AOR], 3.20; 95% CI, 1.18-8.68), and officers who were divorced, widowed, or separated or a member of an unmarried couple had higher odds of a lifetime mental illness diagnosis compared with married officers (divorced, widowed, or separated: AOR, 3.52 [95% CI, 1.35-9.19]; member of an unmarried couple: AOR, 3.64 [95% CI, 1.07-12.33]). Military veteran officers had greater odds of a mental health diagnosis compared with nonveterans (AOR, 3.25; 95% CI, 1.38-7.67). Officers with more than 15 years of experience had greater odds of a mental illness diagnosis (AOR,  7.42; 95% CI, 1.02-54.01), and supervisory staff had more than 3 times the odds of a diagnosis compared with patrol officers (AOR, 3.12; 95% CI, 1.02-9.55).

**Table 3.** Odds of Mental Illness Diagnoses and Positive Screening Results for Mental Illness Symptoms Among 434 Police Officers

Characteristic	AOR (95% CI)	
	Lifetime mental health diagnosis (n = 54)	Positive screening result in past 2 wk (n = 114)
**Sociodemographic**		
Age	0.97 (0.90-1.05)	0.97 (0.92-1.03)
Gender		
Male	1 [Reference]	1 [Reference]
Female	3.20 (1.18-8.68)	1.94 (0.97-3.87)
Race/ethnicity		
Non-Hispanic		
White	1 [Reference]	1 [Reference]
Black	0.04 (0.00-0.35)	0.87 (0.39-1.95)
Hispanic	0.47 (0.19-1.15)	1.43 (0.79-2.58)
Other or >1	NR	0.40 (0.08-1.99)
Marital status		
Married	1 [Reference]	1 [Reference]
Divorced, widowed, or separated	3.52 (1.35-9.19)	1.93 (0.90-4.13)
Never married	1.94 (0.58-6.50)	1.61 (0.78-3.34)
A member of an unmarried couple	3.64 (1.07-12.33)	1.48 (0.60-3.64)
Education		
High school diploma	0.49 (0.08-2.95)	0.31 (0.08-1.16)
Some college or technical school	0.86 (0.39-1.89)	1.07 (0.62-1.86)
College graduate	1 [Reference]	1 [Reference]
Master’s graduate or higher	1.59 (0.27-9.34)	1.45 (0.43-4.90)
Military experience	3.25 (1.38-7.67)	3.46 (1.87-6.40)
**Occupation**		
Length of police service, y		
<5	1 [Reference]	1 [Reference]
5-10	1.86 (0.52-6.60)	3.05 (1.42-6.54)
11-15	4.12 (0.99-17.20)	3.10 (1.18-8.14)
>15	7.42 (1.02-54.01)	1.57 (0.37-6.71)
Shift		
12 pm to 7 am	1 [Reference]	1 [Reference]
7 am to 3 pm	0.64 (0.23-1.79)	0.69 (0.33-1.45)
3-11 pm	0.62 (0.23-1.65)	0.80 (0.42-1.53)
Other	1.17 (0.25-5.46)	0.99 (0.32-3.06)
Rank		
Patrol officer	1 [Reference]	1 [Reference]
Sergeant, lieutenant, or higher	3.12 (1.02-9.55)	0.91 (0.34-2.41)

Abbreviations: AOR, adjusted odds ratio; NR, not reported.

After adjustment for confounders, the odds of a positive screening result were significantly higher among officers with military experience (AOR, 3.46; 95% CI, 1.87-6.40) and officers with 5 to 10 years (AOR, 3.05; 95% CI, 1.42-6.54) and 10 to 15 years (AOR, 3.10; 95% CI, 1.18-8.14) of police service compared with officers with less than 5 years of experience.

Table 4 gives the association between a positive screening result and interest in using mental health care services. After adjustment for confounders, officers with suicidal ideation or self-harm were significantly more likely to be interested in getting help compared with officers who did not report suicidal ideation or self-harm (AOR, 7.66; 95% CI, 1.70-34.48).

**Table 4.** Odds of Interest in Mental Health Care Services Among Officers With a Positive Screening Result for Mental Illness Symptoms Among 114 Police Officers

Variable	OR (95% CI)	
	Crude	Adjusted^a^
Anxiety	3.22 (1.19-8.72)	3.58 (0.79-16.23)
Depression	1.68 (0.68-4.11)	0.78 (0.21-2.85)
Suicidal ideation or self-harm	3.81 (1.38-10.51)	7.66 (1.70-34.48)
PTSD	0.45 (0.12-1.73)	0.60 (0.02-20.01)

Abbreviations: OR, odds ratio; PTSD, posttraumatic stress disorder.

^a^ Models adjusted for age, sex, educational level, military experience, shift, length of service, and relationship status.

Four themes for lack of mental health care use were identified during focus groups, which included (1) inability of officers to identify when they are experiencing a mental illness, (2) concerns about confidentiality, (3) belief that psychologists cannot relate to police stressors, and (4) stigma that officers who seek mental health care are not fit for duty. Evidence for each theme is included in Table 5.

**Table 5.** Perceptions of Use of Mental Health Services

Perception	Examples
Lack of knowledge that an officer is experiencing a mental health issue	“The stress level, we get so accustomed to having the stress that that’s the norm for us”; “Numb to it”; “You may not realize how close you are to needing that stuff because a lot of stuff, you go back and forth between you’re at a worse spot, you’re at a better spot. You kinda get used to being like that”; “Yeah, I’m fine. I’ll deal with it. It’s just a bad time and I’ll come back”
Concerns about confidentiality within the department	“It doesn’t mean that it’s confidential and nobody here trusts this department, and if you do, you’re an idiot”; “This place is like a high school. Words get out here and there”; “You didn’t tell anybody. Somebody saw you and so-and-so told so-and-so”
Belief that psychologists cannot relate to their occupational duties	“My reaction is ‘So what? Who are you?’”; “I don’t need to talk to somebody that has no clue what I’m trying to say”; “Unless it’s someone here I don’t want to talk to them”; “I don’t want to talk to them. They don’t get it. They don’t know. They don’t understand. They’ll look you square in the eye and say ‘I get it and understand.’ I look at them and I go, ‘No you don’t.’ ‘No you don’t, go back to class, go back to school, go back to where you came from’”
Stigma that officers seeking mental health services are not fit to do their jobs	“I think it’s more so that nobody feels they need to go. When you think about it, well, people that go there, they got something wrong or an issue they can’t handle”; “There’s also the reprisal if you go see a shrink, you’re gonna lose your job, or you’re gonna be labeled as a nutter, so that’s a large part of it, as well”; “I’m an officer I should be able to take care of myself. I should be able to handle anything that’s going on”

## Discussion

To our knowledge, this is the first survey study to analyze mental illnesses, symptoms of mental illness, and mental health care use among officers at a large, urban police department. The findings suggest that mental health is an occupational concern because 12% of officers reported a prior diagnosis of mental illness and 26% screened positive for depression, anxiety, PTSD, or suicidal ideation or self-harm symptoms. Quantitative analyses found that mental illness diagnosis was particularly high among female officers; officers who were divorced, widowed, or separated; and officers with military experience. This finding is consistent with past literature.^9,28,29,30,31^ For example, female officers have been reported to experience unique stressors in police departments,^9^ which may be associated with higher levels of work overload^28^ and burnout.^29^ Furthermore, research has found that lack of social support^30^ and combat experience is associated with police officer mental health.^31^

Of note, 26% of officers had a positive screening result but had no prior mental illness diagnosis. This theme was largely reflected in the focus groups, with participants stating that they became accustomed to the stress and traumatic events at work and became “numb to it.” This is concerning because if officers are unaware of how their work is impacting their mental health, they are unaware that they should seek treatment. Among officers with a current mental illness diagnosis, 35% sought services in the past 12 months, and among officers with a screening result positive for mental illness symptoms, 17% sought mental health services. This finding is inconsistent with past research^16^ that found that nearly 50% of suburban officers with mental illness sought mental health services. This discordance may be attributable to the geographic setting. Suburban occupational exposures and associated triggers for mental illness may be different from those in a larger city. Furthermore, Dallas police officers mainly live outside the city, where access to mental health services is typically more difficult than within urban areas.^32^

Other than the inability of officers to identify when they were experiencing a mental illness, officers did not seek services for 3 additional reasons: (1) concerns about confidentiality in the department, (2) not believing psychologists understood their line of work, and (3) feeling like they are not fit for duty. A smaller, qualitative study in the UK found similar results of officers reporting stigma associated with seeking mental health care and lack of support from line management that hindered officers’ desire to disclose mental distress.^33^

Interventions at police departments appear to be needed to identify officers experiencing mental illness and connect them to care in a systematic way, while addressing their concerns. Such interventions may not only help reduce the rate of undiagnosed and untreated mental illness through connection to services but may be associated with improved work productivity.^16,34^ However, the best practice model to adapt in law enforcement has yet to be determined. Past studies^35,36^ among other populations, such as the military or general public, have tried implementing secondary prevention models, such as the critical incident stress debriefing (CISD), to address PTSD. In the CISD model, 1 week after a traumatic event, a group of survivors were led through a 7-step, 1- to 3-hour debriefing session. However, a meta-analysis found that CISD was not associated with reduced symptoms of PTSD and suggested that the intervention was associated with worse outcomes.^37^ Studies^38,39^ have suggested that a more structured and well-defined program, such as cognitive behavioral programs with 4 to 5 weekly individual sessions and homework assignments, may be associated with better outcomes. Psychosocial first aid and peer support forums are also in their infancy in police literature but have been associated with positive mental health outcomes among law enforcement officers while addressing officers’ mistrust of psychological services.^40,41,42,43,44^

Our results indicate PTSD is not the only mental illness among law enforcement. The findings suggest that depression, anxiety, and suicidal ideation or self-harm, which typically take longer to manifest, also should be systematically addressed. A proactive and systematic primary prevention model appears to be needed to identify developing mental illness among officers. For example, an outside, independent agency of mental health care practitioners could potentially screen for mental illness in the police academy and then on an annual basis. This approach may reduce stigma by normalizing the practice of mental health checkups and may offer officers a consistent connection to a list of departmental and nondepartmental services. Nonetheless, we believe that mental health programs should be structurally implemented and rigorously evaluated at police departments to evaluate their association with depression, anxiety, PTSD, and suicidal ideation.

### Limitations

This study has limitations. First, participants were included from only 1 police department; thus, external validity is limited. However, the rigorous methods of data collection allow for generalizability to the entire patrol department. Second, given the time restraint of the survey, we included only the brief screeners of mental illness symptoms. This approach restricted our ability to classify symptoms to clinically relevant categories, which may be a better indicator of the interest and use of mental health services. However, the short versions have high sensitivity and specificity for mental illness in primary care. Third, the sample was significantly different demographically from the general patrol officer population; therefore, it is unclear whether our findings are generalizable to the department and other law enforcement agencies.

## Conclusions

In the present study, although few officers sought treatment, some were interested in seeking help, particularly those with suicidal ideation or self-harm. Future studies should aim to achieve a more nuanced understanding of the types of treatment sought by officers, which would be helpful to inform innovative care delivery strategies that are tailored to officers in the future. Future interventions and, potentially, broader screening policies in law enforcement agencies appear to be needed to systematically identify and refer officers to health care services while mitigating their concerns, such as potential breach of confidentiality.
