# Error in the Abstract Results

**DOI:** 10.1001/jamanetworkopen.2020.29188

**Published:** 2020-11-16

In the Original Investigation titled “Prevalence of Mental Illness and Mental Health Care Use Among Police Officers,”^1^ published on October 7, 2020, there was an error in the second sentence of the Abstract Results. A total of 19 of 114 officers (17%) who had positive screening results for current mental illness symptoms had sought mental health care services in the past 12 months, not 19 of the 434 officers who completed the survey. This article has been corrected.
